# Atomic force spectroscopy‐based essay to evaluate oocyte postovulatory aging

**DOI:** 10.1002/btm2.10294

**Published:** 2022-06-09

**Authors:** Alice Battistella, Laura Andolfi, Michele Zanetti, Simone Dal Zilio, Marco Stebel, Giuseppe Ricci, Marco Lazzarino

**Affiliations:** ^1^ CNR‐IOM SS Trieste Italy; ^2^ University of Trieste Trieste Italy; ^3^ IRCSS Burlo Garofalo Trieste Italy; ^4^ Department of Medicine, Surgery and Health Sciences University of Trieste Trieste Italy

**Keywords:** AFM measurements, IVF, oocytes mechanical properties, postovulatory aging, reproductive medicine, zona hardening

## Abstract

Postovulatory aging is a process occurring in the mature (MII) oocyte leading the unfertilized ones to apoptosis. The optimal time window of fertility for different mammalian species after oocytes maturation depends on its timeliness: the higher the time elapsed from the accomplishment of the MII stage, the lower are the chances of fertilization and of development of a viable embryo. In the in vitro fertilization, the selection of competent oocytes for intracytoplasmic sperm injection (ICSI) is mostly made by the visual inspection of the MII oocyte morphology, which does not allow to determine the oocyte postovulatory age. On the other hand, more specific tests usually involve some kind of staining, thus compromising the viability of the oocyte for reproductive purposes. Hence, the need of a noninvasive analysis of oocyte aging to improve the success rate of in vitro fertilization procedures. Here, we exploit atomic force microscopy to examine the evolution of the mechanical properties of mouse oocytes during in vitro postovulatory aging. Three hours before the occurrence of any visual morphological feature related to degradation, we observe a sudden change of the mechanical parameters: the elastic modulus doubles its initial value, while the viscosity decreases significantly. These mechanical variations are temporally correlated with the release of the cortical granules, investigated by fluorescence microscopy. Interestingly, the oocyte mechanics correlates as well with the yield of embryo formation, evaluated up to the blastocyst formation stage. These results demonstrate that minimally invasive mechanical measurements are very sensitive to the aging of the oocyte and can be used as a label‐free method to detect the age of the postovulatory oocytes.

## 1 INTRODUCTION

The pool of oocytes, with which a female mammal is provided, starts the meiotic maturation during fetal development. At birth, oocytes are arrested at the first meiotic prophase and are characterized by the presence of the germinal vesicle (GV). The nuclear envelope breakdown occurs only at the time of puberty when oocytes enter the first meiotic division (MI). At ovulation, mature oocytes (MII) are released from the ovarian follicle. After release, in the absence of fertilization, the mature oocytes go through a process of slow degradation culminating in apoptosis.

This phenomenon is called “postovulatory aging” and its duration varies according to species: in humans, the useful time window for fertilization is lower than 24 h, while in mice is lower than 8–12 h.^1^ The oxidative stress is thought to be the main responsibility of the aging processes, which leads to different events: a decreased level of the maturation promoting factor (MPF, a cellular factor involved in maintaining the cell cycle on idle); the mitochondrial dysfunction related to the damage of mitochondrial DNA and membranes; the impairment of Ca^2+^ channels and ATPases that affects Ca^2+^ homeostasis, and variations in the dynamics of actin‐tubulin assembly that are related to the formation of the meiotic spindle.^2^, ^3^

A further key event related to postovulatory aging is the occurrence of a spontaneous cortical granule (CG) exocytosis.^1^ CG exocytosis is a reaction that normally occurs as a direct consequence of fertilization, and it involves the release of ovastacin, a protease that cleaves and cross‐links the ZP2, one of the three glycoproteins of the zona pellucida (ZP).^4^ This event causes considerable changes in the ZP structure: the thickness of the fibrils increases and the mechanical properties change as shown by the increased stiffness and proteolytic resistance after the fertilization.^5^ These modifications help to avoid the occurrence of polyspermy by hindering the penetration across the ZP of an excess of spermatozoa.^6^, ^7^

The clinical implications of postovulatory aging include decreased rates of fertilization, as it occurs in “rescue” ICSI performed on failed‐to‐fertilize oocytes, loss of the embryo uterine implantation capacity, and impaired development during embryogenesis. Moreover, dysfunctional mitochondria and altered epigenetic profile can cause abnormalities in offspring (growth retardation, decreased reproductive fitness, and longevity).^8^, ^9^

To increase the number of competent oocytes during the in vitro fertilization (IVF) practice, women are treated with different gonadotropin stimuli to induce superovulation; therefore, the retrieved oocytes can have different maturation stages. The embryologist selects competent MII oocytes looking at those visual morphological features that are commonly related to mature healthy oocytes. The presence of the first polar body (PB) identifies a meiotically mature MII oocyte, while an immature oocyte is characterized by the absence of PB (MI) or the presence of the germinal vesicle (GV), the egg nucleus with the condensed chromosomes. The regular structure and thickness of the ZP, and the absence of cytoplasmic granularity are some of the key parameters associated to a good quality oocyte.^10^, ^11^ While the maturation phase of an oocyte can be identified with the help of an optical microscope, the postovulatory age has no visible morphological features. Visual inspection allows to separate apoptotic oocytes from those still showing “healthy features,” but among them only a few have the potential to generate live birth. Often, fertilization does not occur, and the embryo development stops at the first divisions, or the blastocyst fails to adhere to the endometrial tissue. As the precise moment of the meiosis start cannot be known, it should be considered that oocytes retrieved from the superovulation practice may have different postovulatory ages, and thus different chances of a positive outcome of the IVF practice.

Further analytical approaches beyond visual inspection have been proposed to determine the stage of MII oocyte such as genetic screening, spectroscopy‐based metabolomic profiling, enzymatic activity assays, and protein expression analysis on follicular fluids^12^, ^13^, ^14^ Nevertheless, these methods are either invasive, with the risk of oocyte damage, or expensive, and time‐consuming.^15^ On the other hand, polscope‐based imaging and fluorescence lifetime imaging microscopy (FLIM) measurement of nicotinamide adenine dinucleotide (NADH) and flavin adenine dinucleotide (FAD) commonly referred to as metabolic imaging are safer methods that do not compromise the oocyte viability and chances of fertility,^16^, ^17^ have been proposed, indicating that quick and safe alternative are needed to determine with a better accuracy the MII stage and the age of the oocyte.

Recently, there is a growing attention to the cellular mechanical properties for their relation to the physiological or pathological cell status.^18^, ^19^, ^20^, ^21^ Biomechanical parameters, evaluated through different techniques, have already been proven to be good noninvasive predictors for embryo and zygote viability, as well as for the detection of the maturation phases of the oocytes.^22^ Microtactile sensors and micropipette aspiration of whole oocytes were used to investigate the phenomenon called “zona hardening” after fertilization, which involves a ZP stiffening after fertilization.^23^, ^24^ Indentation measurements performed by atomic force microscopy (AFM) of isolated ZP allowed to identify the contribution of two layers with different mechanical properties, which were observed to be involved in the postfertilization cross‐linking of this glycoproteic membrane.^25^ Likewise, AFM‐based indentation performed on whole human oocytes showed significant differences in ZP stiffness among different stages of oocyte maturation classified by visual inspection (GV, MI, and MII oocytes).^26^ These findings suggest the AFM approach can be particularly attractive for oocyte analysis since it enables a fine control of the applied forces compared to the previously mentioned techniques, resulting in small oocyte deformations.

In this article, we examined the mechanical properties of mature (MII) mouse oocytes by AFM indentation. An alteration of the mechanical features of the aged oocyte can be detected few hours before the morphological changes related to apoptosis become evident by visual inspection. This event is temporally anticipated by a CG exocytosis, which prompts us to assume the occurrence of a CG‐induced cross‐link reaction in the ZP.

This could allow the identification of a more reliable parameter to classify the quality of the oocyte that could be exploited for increasing the yields of IVF.

## 2 RESULTS

### 2.1 Identification of the relevant mechanical parameter

**FIGURE 1:**
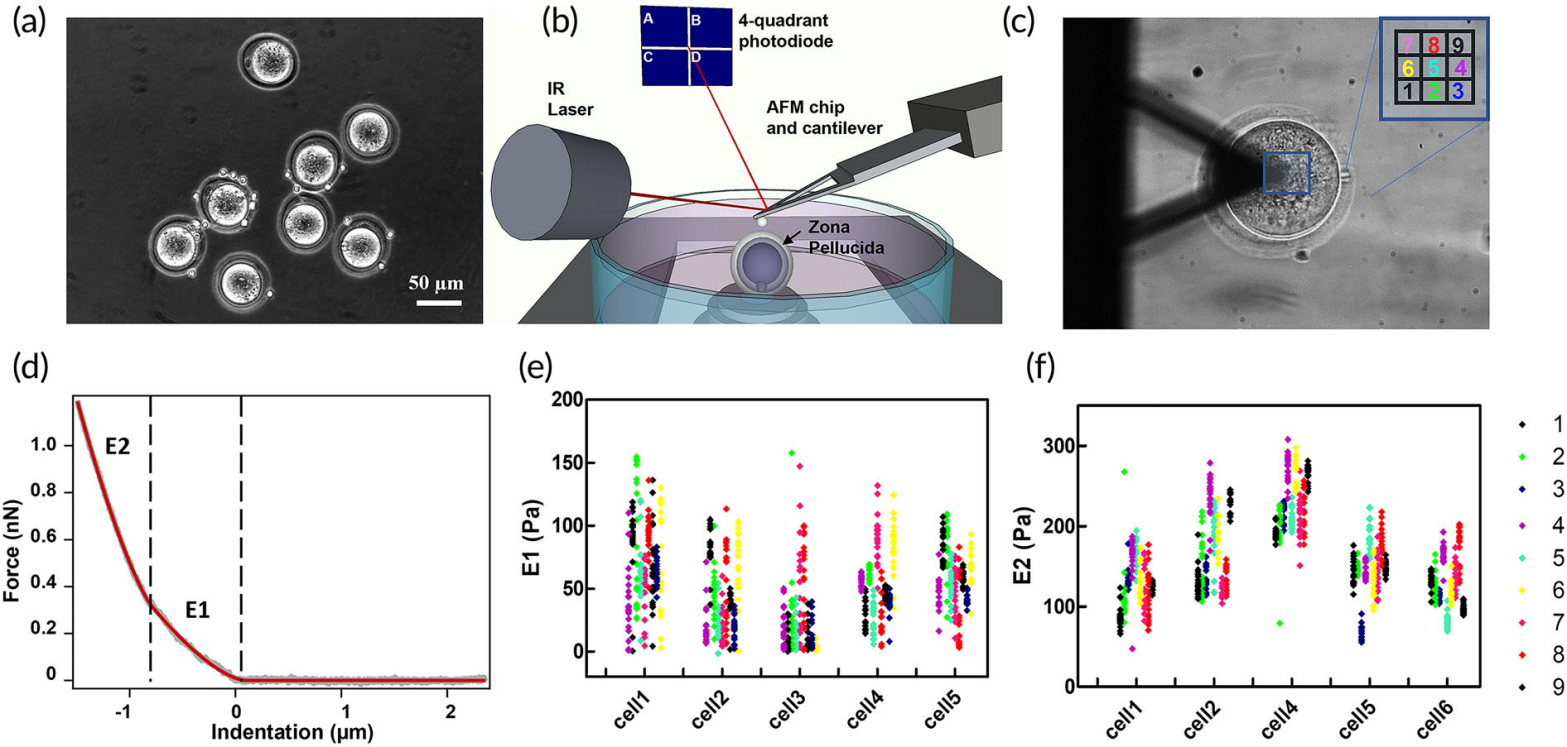
Evaluation of the mechanical properties of mouse oocytes. (a) Pool of MII oocytes retrieved from a mouse after the removal of the cumulus complex; (b) schematic representation of the experimental setup: an oocyte is probed by the cantilever whose deflection is controlled by the position of the laser on the photodiode, ZP, and the first polar bodies are depicted; (c) top‐view of an oocyte during an AFM‐nanoindentation measurement, with beadmounted cantilever approaching at the center of the cell, measurements are performed in a squared area of 3 × 3 μm; (d) fitting of the force‐distance curve according to the two contribution model, the two slopes are shown resulting in two values of the Young modulus (*E*1 and *E*2); (e) E1 values distribution for five representative cells, the colors correspond to different positions of the cantilever on the cell. *E*1 values distribution are larger within the same cell than on different cells, this does not allow to appreciate position and cell heterogeneity; (f) *E*2 values distribution within the same cell is small enough that values distribution changes according to the probed area and the difference between the mean values of individual cells can be appreciated. AFM, atomic force microscopy; MII, mature oocytes; ZP, zona pellucida

To identify the suitable mechanical parameters to study the oocyte aging, we investigated the mechanical properties of the whole mouse oocyte by performing AFM indentation measurements on a population of denuded MII mouse oocytes retrieved from superovulating mice (Figure 1a). We used tipless triangular cantilevers with 5‐μm beads glued at their ends that were approached to a small region on the top of the cell (Figure 1b).

To evaluate the role played by the local inhomogeneities that characterize the outer layer of the ZP, the AFM indentation was performed on a 3 μm × 3 μm grid as shown in Figure 1c. The analysis of force‐indentation curves highlighted the presence of two slopes similarly to what was observed for human oocytes^26^; therefore, we used a fitting procedure based on a modified Hertz model to take in account the sum of two contributions. As a result, two elastic components were identified: a first Young's modulus (E1) related to the outermost layer of the ZP, and a second Young's modulus (*E*2) associated with the more internal part of the ZP (Figure 1d). Side‐view imaging of the indentation process (Figure S1) highlighted that the contribution of the inner components of the oocyte such as the perivitelline space (PVS) and the ooplasm, if present, is negligible. The resulting *E*1 and *E*2 values obtained for each point of the grid are reported for several oocytes in Figure 1e. Within the same oocyte, the *E*1 values are scattered with a variability that can be comparable to the variability from cell to cell. Due to the micrometric irregularities of the surface of the oocyte, the presence of glycoprotein protrusions, and the porous mesh‐like structure of the mature oocyte ZP (see SEM image in Figure S2) the position of the first contact point is not well defined. Moreover, the outermost part of the glycoprotein layer is not uniform, this can contribute to a wide distribution of the *E*1 values within the same oocyte which, in turn hinder the use of this parameter to produce meaningful discrimination between different cells. On the contrary, the *E*2 values obtained on different positions of the same cell resulted narrowly distributed (Figure 1f). These observations suggested the choice of *E*2 as a reliable and sensitive parameter to investigate the evolution of the mechanical properties of ZP and the oocyte upon the postovulatory aging process.

Moreover, to increase the sample size by reducing, at the same time, the number of sacrificed animals, we investigated the effect of the freezing procedure on mice oocytes. Oocytes were frozen according to the protocol described in the supplemental material. The *E*1 and *E*2 values were evaluated for freshly retrieved oocytes and for oocytes subjected to a freeze–thaw procedure (Figure. S3). To compare properly the two populations, for the former, mechanical measurements were performed within 1 h from the oocytes retrieval, while for the latter measurements were acquired measured within 1 h from the thawing. The mean *E*1 and *E*2 values of thawed oocytes are, respectively (50 ± 5) and (160 ± 10) Pa and are comparable with those obtained for freshly retrieved oocytes, respectively (43 ± 4) and (148 ± 7) Pa in agreement with what observed for human oocyte.^27^ These data confirmed that the freeze–thaw procedure does not affect the mechanical properties of the oocyte. Therefore, both freshly and thawed oocytes were used to investigate the postovulatory aging process).

### 2.2 Does the AFM indentation trigger postovulatory aging?

**FIGURE 2:**
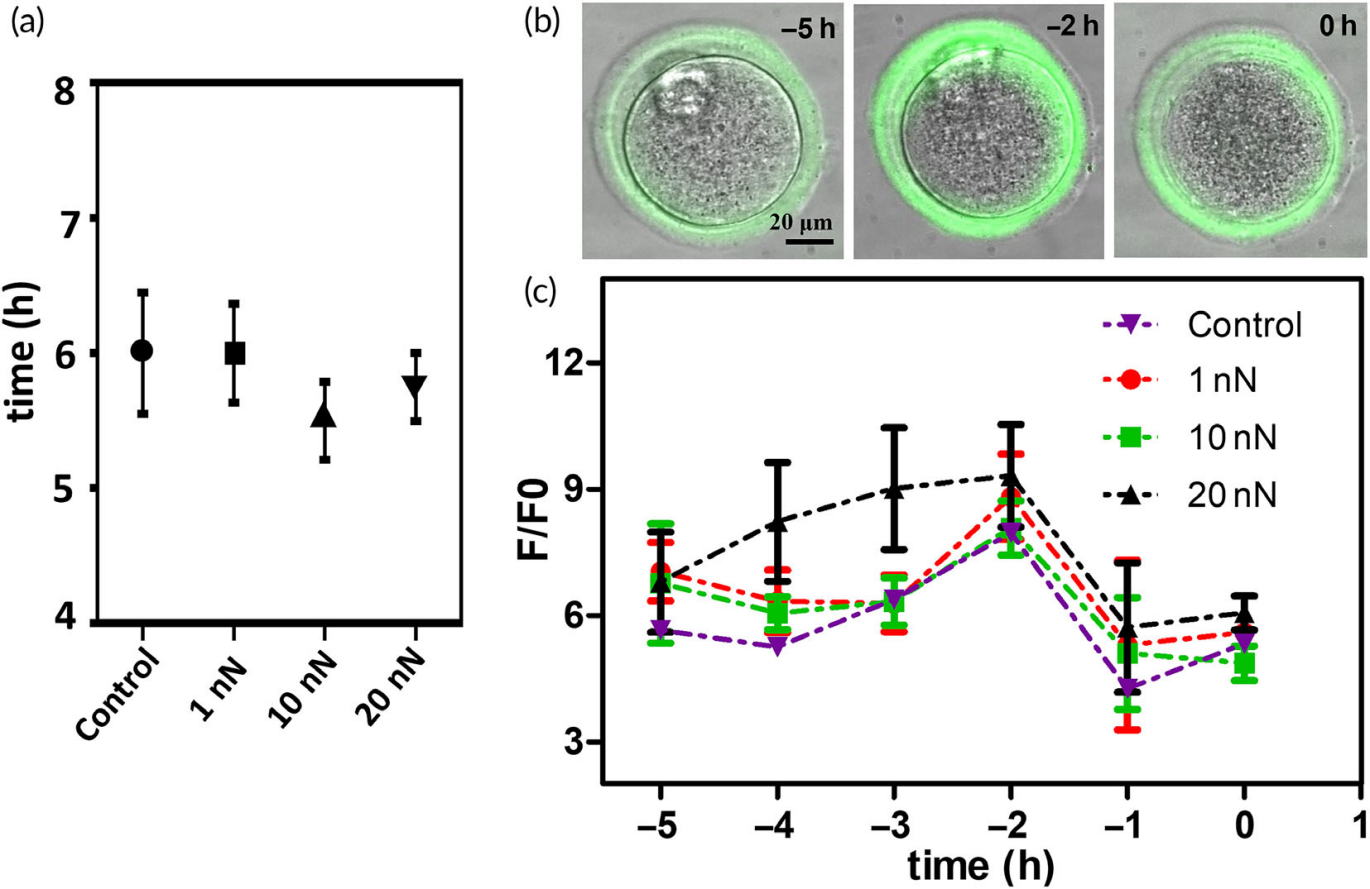
Effect of different mechanical loading on the oocyte postovulatory aging progression. (a) mean values and *SD* of the postovulatory oocyte lifespan in three groups of oocytes in which different force loading were applied (1, 10, and 20 nN), and the control group in which no force was applied (*N* = 6, for each condition). (b) Merged images of bright field and fluorescence show the variation of the oocyte morphology related to aging and the increase in the intensity of the fluorescence related to the CG release about 2 h before the occurrence of the visual degradation. (c) Variation of the fluorescence signal in time during CG exocytosis when different mechanical stresses 1, 10, and 20 nN are applied at three groups of oocytes and in the absence of applied force (control). To compare different oocytes with a different stage of CG exocytosis, the occurrence of visual degradation was chosen as zero of the time scale. Accordingly, the data acquired before the degradation were plotted on a backward time line. The mean values and the standard error are showed for each time point (*N* = 5, for each condition). CG, cortical granule

To rule out the possibility that the indentation measurements themselves can have an effect on the dynamics of the postovulatory aging, we used cryo‐conserved oocytes to investigate is the possibility of a correlation between degradation time and indentation force. Indeed, the mean elapsed time between thawing and the onset of the visually detected morphological alterations did not change significantly, and all the tested conditions were similar to the control group, where no force was applied (Figure 2a).

To better monitor in‐vitro the effect of the AFM indentation on the health and postovulatory evolution of freshly retrieved oocytes, we investigated the correlation between CG exocytosis and the force applied performing the AFM indentations. For this purpose, we added to the culture medium a solution of FITC‐LCA (FITC‐LCA is a fluorescein conjugated lectin [FITC is the fluorescein and LCA stands for lens culinaris agglutinin, a specific lectin]), a lectin‐conjugated fluorophore that binds to the α‐mannose residues which are released by GCs during the exocytosis process. Initially, a background fluorescence can be observed everywhere; after the occurrence of spontaneous GC exocytosis, the α‐mannose concentration in the PVS peaks, and so do the bound fluorophores, resulting in a weak, but detectable, local increase of the fluorescence, as shown in Figure 2b.

Then, different forces were applied on the oocytes by AFM with a bead‐modified cantilever. Starting from the thawing time, up to oocyte visible degradation, forces of 1, 10, and 20 nN were applied to different oocytes groups, and epifluorescence images were taken every hour. During this interval, the oocytes were kept at 37°C in the incubator (5% CO_2_) and extracted only for the time needed for image acquisition. Their morphology was monitored by bright field microscopy, considering healthy specific visual features, such as the presence of a round‐shaped ooplasm, a regular ZP, and the presence of the PB. Oocyte degradation was identified when anomalous morphological features appeared, such as an increase in cytoplasm granulometry, a visual deformation of the ooplasm, and the oolemma break down resulting in the disappearance of the PVS.^28^

In Figure 2b, a merging of bright field and fluorescence images of the same oocyte at three different aging times is shown. To compare the aging‐related fluorescence variations of different oocytes, the occurrence of visual degradation was chosen as the time reference zero, to which data from different oocytes are aligned. As a result, the data acquired before the degradation are plotted on a negative timeline: the time before degradation.

The oocyte in vitro evolution, as monitored by granules exocytosis, observed at different applied forces and in the absence of the applied force is shown in Figure 2c. Approximately 2 h before any visual morphological changes related to oocyte degradation, we observed that the CG‐related fluorescence signal increased both in the absence and presence of applied forces, with a smooth broadening in time of the GC response when a force of 20 nN was applied. From these data, we can draw two main conclusions. First, the application of forces as high as 10 nN do not affect CG exocytosis and do not induce premature in vitro oocyte aging. Therefore, building on these results, we decided to use a 1‐nN force setpoint for the postovulatory in vitro mechanical assay, to operate in a safe condition with minimal stress for the oocyte.

Second, the CG exocytosis occurs 2 h before the manifestation of any early visual indication of oocyte degradation. Unfortunately, this information cannot be applied to the evaluation of oocyte aging within IVF protocols since it requires the use of chemicals that can affect embryo development and the newborn health.

### 2.3 The mechanical properties of the oocyte depend on the postovulatory aging

The postovulatory mechanical properties of both freshly retrieved and cryo‐preserved oocytes were evaluated by AFM indentation measurements as a function of aging time. Measurements were taken at 1‐h intervals, while between two subsequent measurements, the oocytes were kept under incubator conditions and taken out for approximately only 10 min for each measurement.

At the beginning, fresh and thawed oocytes were separately investigated. As the trend of the *E*2 variation during the time of postovulatory aging was consistent among these two groups (Figure S4), the data are treated as a single population and merged.

**FIGURE 3:**
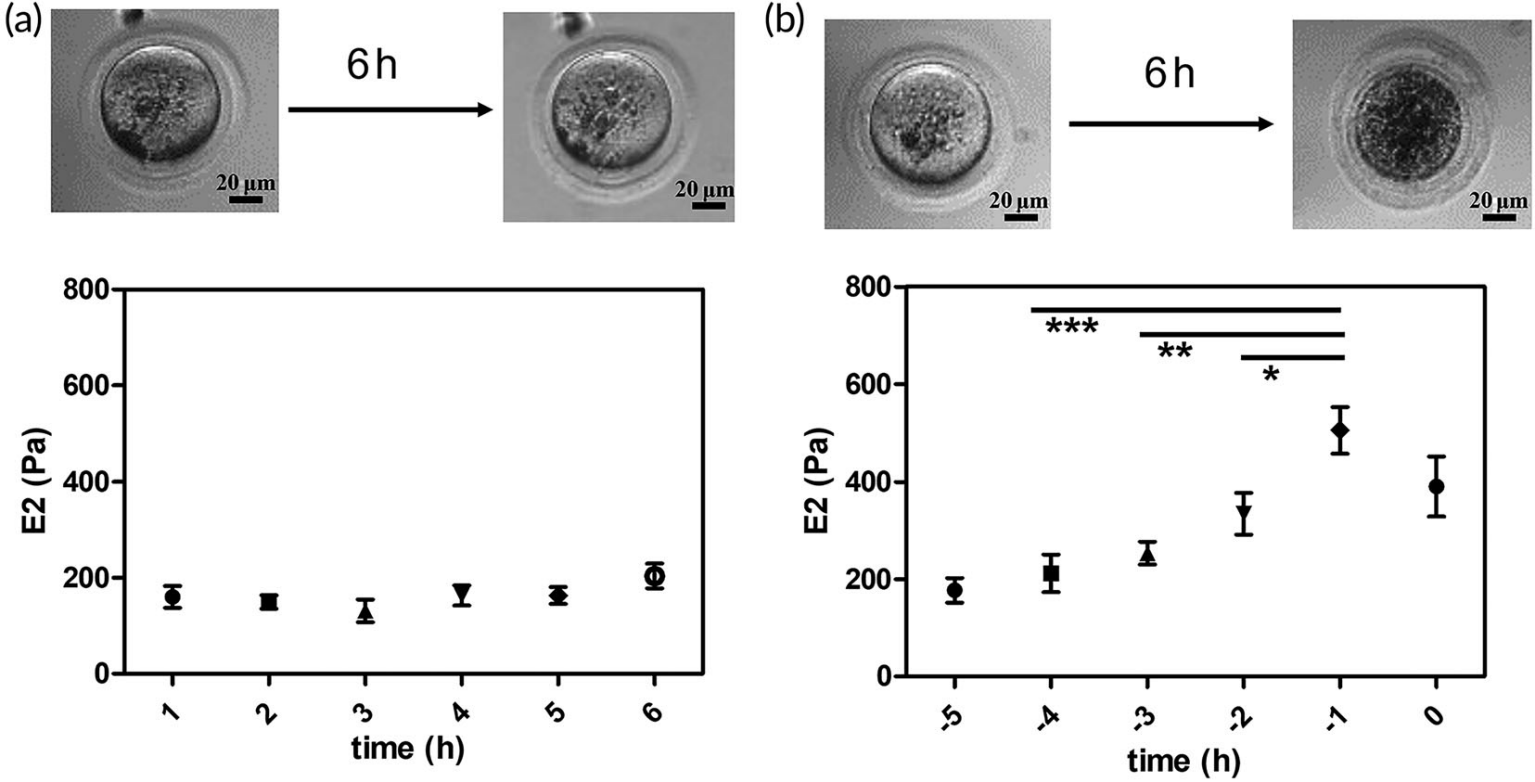
Stiffness variation according to the oocyte postovulatory aging. (a) Oocyte morphology and *E*2 variation during in vitro aging for oocytes that did not reach degradation during the observation period (up to 6 h from the retrieval/thawing). The variation is not significative (*p* > 0.05). Time scale is numbered in hours from the retrieval, which could not account for the aging before and during oocyte extraction (*N* = 15). (b) A live oocyte is characterized by a clear moderately granulate cytoplasm and a round‐shaped ooplasm. After 6 h, oocyte degradation occurs. Visual features related to cell death are a brown cytoplasm and an indistinct plasma membrane. *E*2 variation is showed during in vitro aging for oocytes that reached degradation during the observation period (up to 6 h from the retrieval/thawing). Again, visual inspection does not provide information on the aging before the retrieval, but the degradation time is now well defined. Data of each cell are aligned on the time of degradation and plotted against the time scale of the time before degradation. A significative increase in ZP stiffness is observed 1 h before the visual degradation (*N* = 20). The significance level was set at *p < 0.01, **p < 0.001, and ***p < 0.0001. ZP, zona pellucida

According to their morphological features, the oocytes were divided into two groups: those that maintain the “healthy morphology” 6 h after the retrieval/thawing and those that during the experiment reached a visible state of degradation (Figure 3). Indeed, although we adopted the same procedures and the same time scale for all the investigated oocytes, such as the age of the mice, the time from the hormonal treatment, and the surgical retrieval (whose duration may differ not more than 10 min from day to day), different MII oocytes showed significantly different degradation times.

**FIGURE 4:**
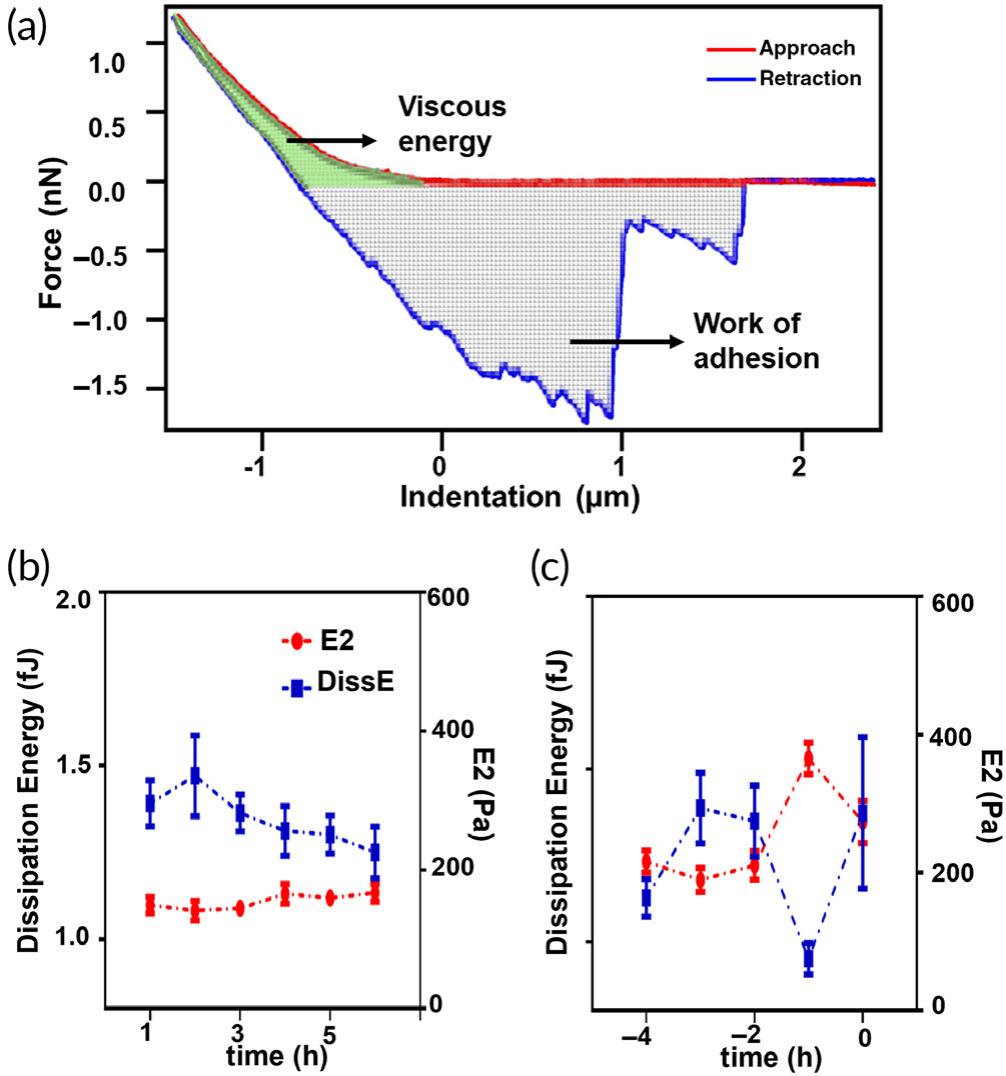
Viscosity variation according to the oocyte postovulatory aging. (a) Force‐indentation curve, the area between the approach and the retraction corresponds to the dissipation energy, the part highlighted in the selection is related to the viscous energy dissipated during the compression of the sample by the tip; (b) the trend of two parameters is shown during in‐vitro aging, for oocytes that did not reach the degradation during the observation period; and (c) for oocytes that reached the visual degradation during the observation time. While the stiffness (represented by the variation or *E*2 in red) increases, the dissipation viscous energy decreases and vice‐versa

The average values of *E*2 measured every hour for the two oocyte groups are shown in Figure 3. As for the data shown in Figure 2, to compare the aging‐related stiffness variation of different oocytes, the data acquired before the degradation were plotted as “time before degradation,” choosing the occurrence of visual degradation as the zero of the time scale. We first observe that, for the oocytes that preserve healthy morphological features characterized by a clear moderately granulated cytoplasm and a round‐shaped ooplasm, no changes in stiffness can be detected (Figure 3a). On the contrary, the oocytes with features associated with a degraded status characterized by a brown cytoplasm and an indistinguishable plasma membrane at the end of the observation period show significant stiffness variation before degradation (Figure 3b). Specifically, before any visual morphological change can be observed, a sudden increase of the *E*2 values occurs, which is immediately followed by a lowering of the *E*2 value. In addition to the Young's moduli values, the dissipated viscous energy was calculated by integrating the area between the approach and retraction curve in the positive force region as shown in Figure 4a. This allows to estimate the viscous contribution during the compression process.^29^ In Figure 4b, the dissipation energy and the *E*2 values plotted as function of the time before degradation show an opposite behavior: 1 h before the visual degradation, the energy dissipated during indentation shows a sudden decrease. These data together are consistent with the occurrence of a cross‐linking of the ZP glycoproteins, similarly to the reaction that occurs in the egg after the fertilization.

### 2.4 Oocyte rheological properties follow the same dependence on the postovulatory aging

**FIGURE 5:**
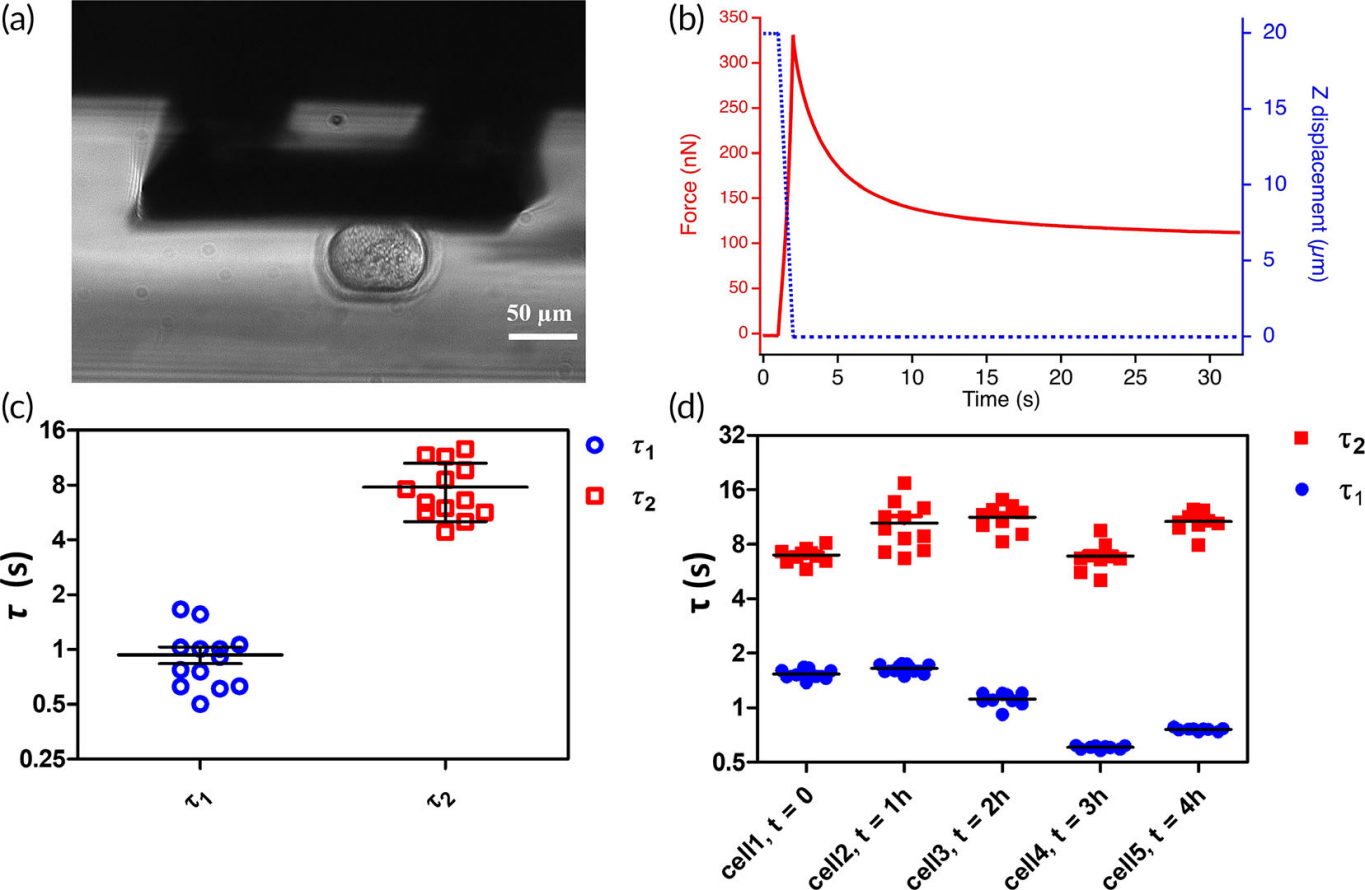
Stress‐relaxation measurement and relaxation time variation during the oocyte postovulatory aging. (a) Side view of the oocyte compressed by the macro‐probe, (b) stress‐relaxation measurement of an oocyte, in which a deformation of 20 μm is applied for 30 s (displacement is depicted in blue). During this time the force exerted by the cell on the probe could be measured (in red), (c) relaxation time (*τ*_1_ and *τ*_2_) mediated for five oocytes are shown, *τ*_2_ (in red) being consistently higher than *τ*_1_ (in blue), (b) the variation in time during oocyte postovulatory aging of these two parameters for five cells. Each point here corresponds to a single measure

To further confirm that the observed effects are not linked to local properties but reflect the status of the whole oocytes we performed whole‐oocytes stress relaxation measurement using macrocantilevers fabricated on purpose.^30^ The side view of a compressed oocyte is shown in Figure 5a. Stress‐relaxation measures the decrease in time of the force that the sample opposes to an external strain (Figure 5b), and the dynamics of this change depends on the ratio between the sample viscosity and sample stiffness. The lower viscous and the stiffer the sample, the faster is the reaction. According to the local indentation data discussed in the previous paragraph, if reported to the whole cell, we would expect to observe the halving of the relaxation time 1 h before the degradation. Therefore, we performed whole‐cell relaxation experiment on five oocytes at different times from retrieval. We observed that the stress‐relaxation curve is described by at least two Maxwell elements in series, each with a different time relaxation constant ($\tau_{1}$ and $\tau_{2}$), as depicted in Figure 5c. Differently from indentation measurements, these stress‐relaxation measurements involve both the ZP and the ooplasm, and it is reasonable to assume that each of the two time constants is related to either the former or the latter, but the proper assignment is not trivial. To clarify this issue, we measured the Young's modulus of the ooplasm, mechanically deprived by the ZP. The same oocyte was measured by AFM‐indentation before and after the ZP removal. The dissipated viscous energy was also calculated and the mean values for these different oocyte compartments are reported in Figure S5, where two representative force‐distance curves are also shown. The difference between the two slopes is reflected by the distribution of the Young's moduli: *E*2 of the ZP‐surrounded oocytes (ZP‐Oo) is significantly higher than that of the Ooplasm (Oo), (280 ± −80) and (90 ± 30) Pa, respectively. At the same time, the energy dissipation is much lower on ZP‐Oo than on Oo, indicating that the ooplasm is a more viscous and softer structure.

These observations helped us to assign the faster relaxation time, $\tau_{1}$, to the ZP and $\tau_{2}$ to the ooplasm, which relaxes over a longer time interval. With this assignment, the decrease in time of $\tau_{1}$ 3 h after the retrieval (Figure 5d) reproduces what we previously observed with AFM‐indentation: during postovulatory aging, the ZP becomes stiffer and the viscosity decreases, consistently with the occurrence of a cross‐link of this layer. In conclusion, we confirm that the evolution of the mechanical properties of the oocytes has a general value and can be used as an indicator of postovulatory aging and that they can be evaluated alternatively measuring the Young's modulus by AFM‐based local indentation, or measuring the relaxation time by whole oocyte stress relaxation investigation, or with any other mean, such as microfluidic or microrheological essays, with equivalent significance.

### 2.5 The mechanical properties of the oocytes depend on the release of the GC

**FIGURE 6:**
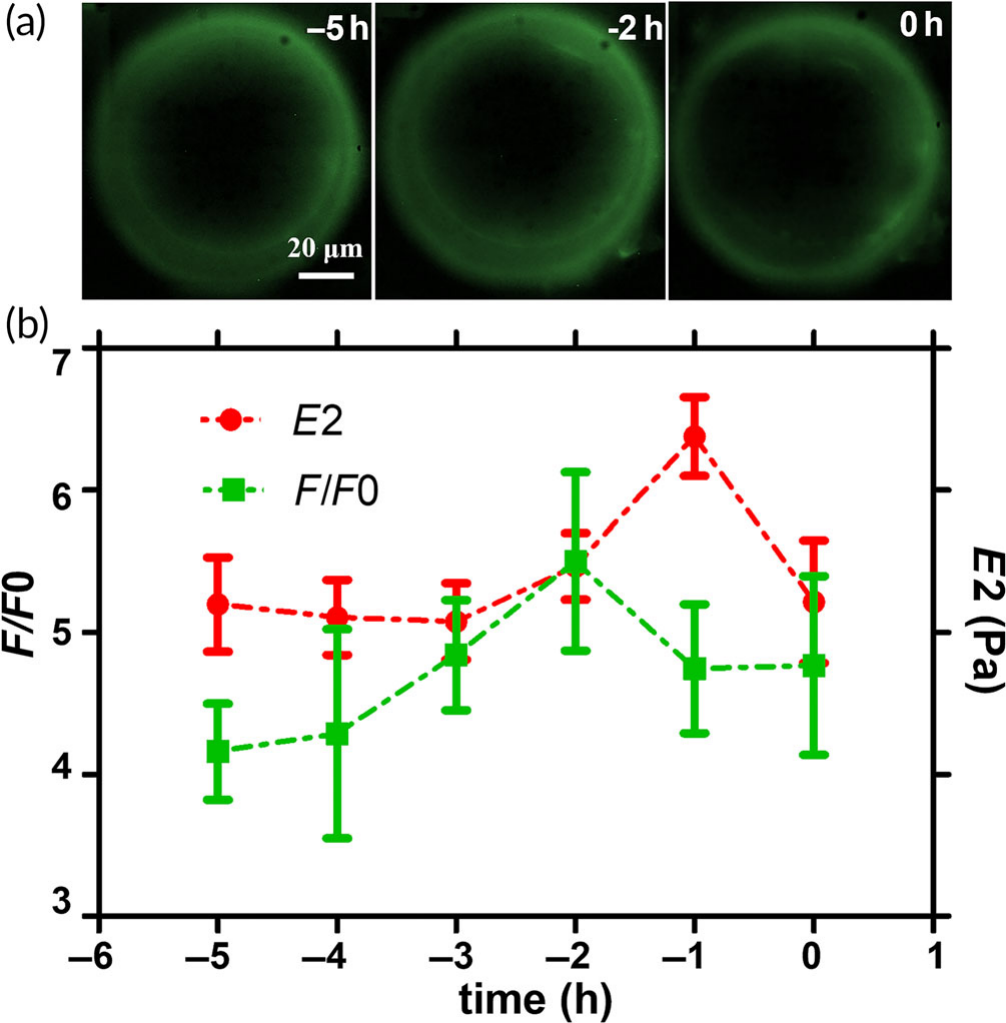
Simultaneous evaluation of stiffness variation and CG exocytosis. (a) the variation of the fluorescence intensity CG related is shown at three different times before the occurrence of the visual degradation; (b) the same cells (*N* = 25) were probed for the FITC‐LCA intensity signal (in green) and the *E*2 variation in time (in red), the results show a progressive increase in the stiffness of the aged oocyte that is anticipated by an increase in the fluorescence related to the CG release. CG, cortical granule

To understand whether the release of GC is involved in the observed increase in stiffness, we repeated the AFM indentation measurements in combination with epifluorescence microscopy to monitor GC exocytosis during in vitro oocyte aging. In Figure 6a, we display the epifluorescence image of the same oocyte at three different aging times, while fluorescence intensity and the *E*2 values recorded at 1‐h intervals until oocyte degradation are shown in Figure 6b. An increment in the fluorescence intensity was detected about 1 h before the variation of *E*2. These data suggest that the ZP stiffening in the oocyte just before degradation may be caused by the CG exocytosis which happens naturally anticipating by roughly 1 h the observed aging‐induced zona hardening. As regards the factor responsible for this reaction, the ovastacin‐mediated “zona hardening” is one of the hypothesized mechanisms that usually takes place in hours from fertilization. Other faster and reversible reactions occurring at the level of oocyte ZP have only been postulated, the main hypothesis being related to the release of some molecules or ions (as Zn^2+^) able to directly induce a fast crosslinking of this glycoproteic layer.^31^

### 2.6 ZP Stiffness variation in parthenogenetically activated oocytes

To further understand the effect of CG exocytosis on ZP mechanical properties, we chemically induced the CG exocytosis adding to the medium a well‐known parthenogenetic activator, SrCl_2_. The main effect of SrCl_2_ is the Ca^2+^ release from the internal compartments of the cell. Ca^2+^ oscillations involve the activation of the meiotic resumption and eventually the formation of the two pronuclei of the parthenogenetic embryo. This chemical activator was already proved to also elicit the cortical granule reaction in the MII oocyte.^32^

**FIGURE 7:**
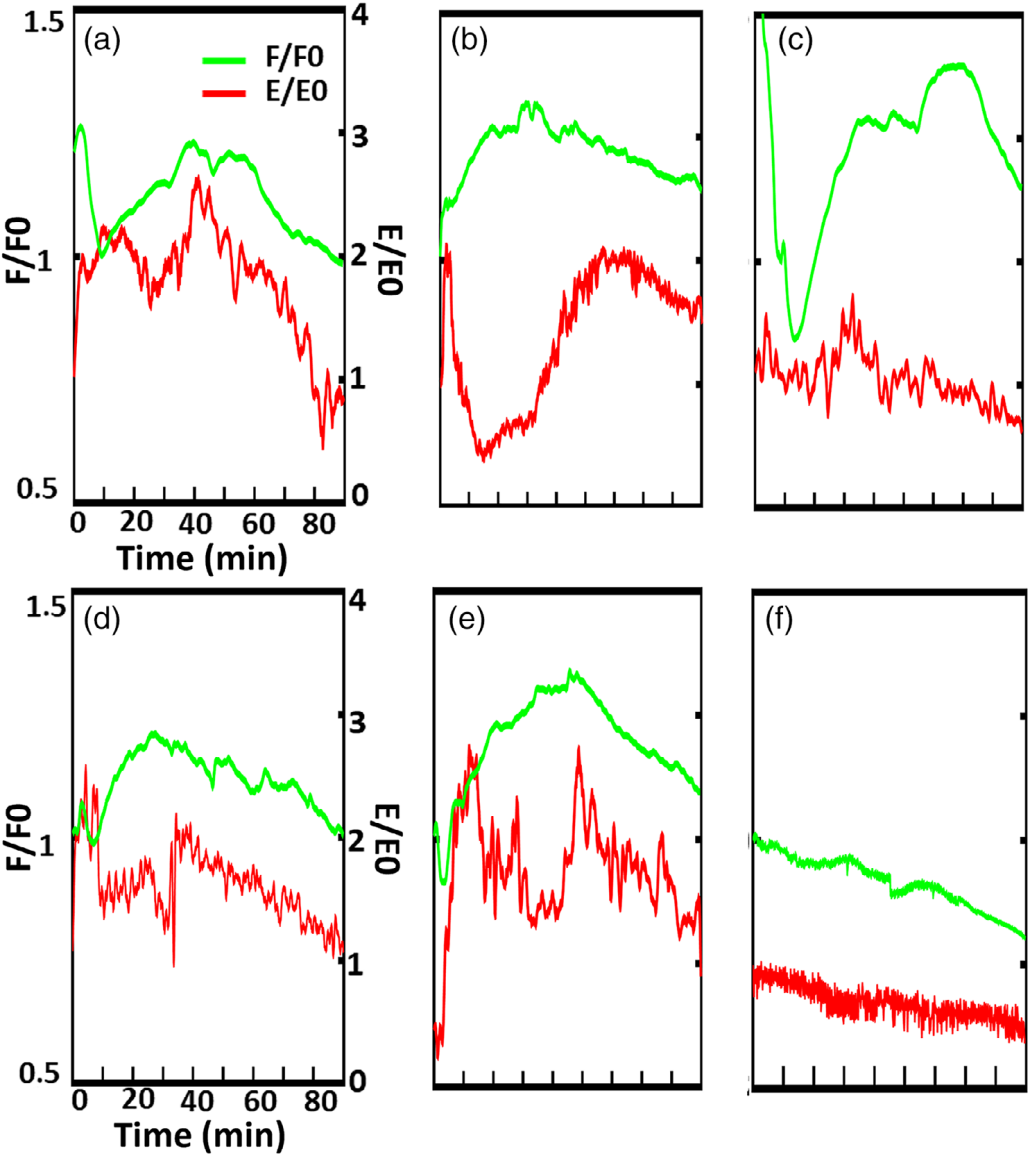
Stiffness variation and CG release evaluation after parthenogenetic activation. After addition of the SrCl_2_, the intensity of the fluorescent signal related to the CG exocytosis (in green) rapidly increases reaching the maximum intensity after 30–40 min, then progressively decreases; also the second Young modulus (in red) undergoes an increase after the addition of the activator, but the dynamics of this variation are different in 5 different cells (a–e); Negative control: without any chemical activation neither the stiffness nor the fluorescence intensity change in time (f). All Data are normalized to the first value which corresponds to the SrCl_2_ addition

After the incubation with the fluorophore, oocytes were activated with 30 mM of SrCl_2_ and the change in the mechanical properties as well as the variation in the FITC‐LCA fluorescence intensity were continuously recorded for up to 90 min. Few minutes after the addition of the activator, CG exocytosis was detected by a sudden increase of the fluorescence signal, which reached the maximum intensity within 30 min and then it gradually decreased. This behavior was consistently observed for all investigated oocytes. On the contrary, although the activator had a clear influence on the ZP stiffness, a regular and simple behavior was not identified. The activator determined strong oscillation on the stiffness with time, frequency, and intensity strongly dependent on the investigated oocyte, which did not allow to extract a common trend (Figure 7a–e). The negative control (Figure 7f), in the absence of the activator, did not show significant oscillations, neither in the fluorescence intensity nor in the *E*2 values, but only a 20% monotonic reduction in both fluorescence intensity and stiffness. As discussed in Section 4, the effect on the fluorescence could be partially attributed to the fluorophore photobleaching; however, the same effect cannot explain the large and rapid intensity variation induced by the CG stimulated release both in stiffness and fluorescence intensity, which therefore confirm the effect of CG release on ZP mechanical properties.

### 2.7 ZP stiffening in the aged oocytes correlates with a decrease in the IVF yields

To investigate how the observed ZP stiffening affects the ability of the oocytes to be fertilized, oocytes were thawed and measured by AFM‐indentation. Two groups of oocytes were analyzed: the first group was measured after 1 h from the thawing, the second group 6 h later.

**FIGURE 8:**
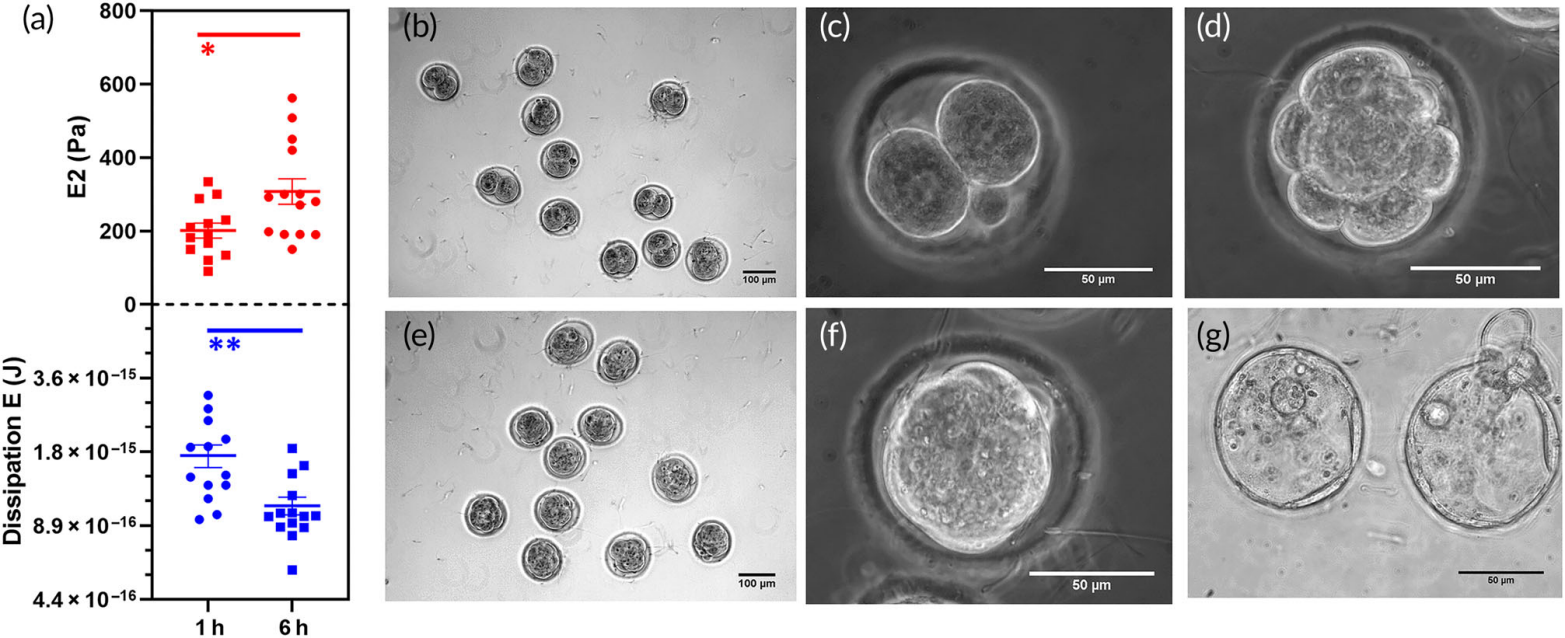
ZP stiffening in the aged oocytes correlates with a decrease in the IVF yields. (a) The variation in Dissipation energy (in blue) and *E*2 (in red) for two groups of oocytes (*N* = 15) 1 h after thawing and 6 h later. There is a statistically significant change in both the mechanical parameters in the two groups of oocytes. The significance level was set at *p < 0.03, **p < 0.002. (b and c) Optical images of 2‐cell embryos, 10× and 40× magnification, respectively. (d) 8‐cell‐stage embryos. (e and f) Early blastocyst, 10× and 40× magnification respectively. (g) Late blastocyst. IVF, in vitro fertilization; ZP, zona pellucida

The values of *E*2 and of the dissipation viscous energy are shown in Figure 8a, in which the mean values and SD are also depicted. After 6 h, we can notice a significative increase of *E*2 in the aged sample compared to the freshly thawed one and a consequent significative decrease in the value of the dissipated viscous energy. Immediately after the measurement, the oocytes were subjected to the IVF procedure. In addition, a “control group” in which the mechanical measurement was not performed was directly fertilized about 1 h from the thawing. The yields of embryo development were evaluated after 24 h (2‐cell embryo stage, depicted in Figure 8b,c). After 72 h, it was possible to observe the embryo development to the 8‐cell stage (Figure 8d), and after 80 h, we evaluated the yields related to the early blastocyst stage (Figure 8e,f). Approximately 15 h later, few of the embryo reached the late blastocyst stage (Figure 8g). When considering the yields of the 2‐cell embryos, we noticed a significative decrease in the 6‐h aged sample: in the group “freshly thawed” oocytes 10 of 14 developed into a 2‐cell embryo (77%), a similar percentage can be found in the control group (80%), while in the “aged” group, only four oocytes of 14 succeeded in reaching this stage (29%). When looking at the development of the blastocyst, seven 14 oocytes managed to reach this stage in the “freshly thawed” oocytes (50%), nine of 15 reached this stage in the control group (80%), while only one of 14 oocytes reached the blastocyst stage in the “aged” oocytes group (7%). The results are summarized in Table 1.

**TABLE 1:** Embryos yields and stiffness variation

Time elapsed from thawing	*N* total	*N* 2‐cell (%)	*N* early blastocyst (%)	*N* late blastocyst(%)	Stiffness (E) (Pa)
1 h	14	10 (77)	7 (50)	3 (21)	201 ± 20
6 h	14	4 (29)	1 (7)	0	310 ± 35
Control	15	12 (80)	9 (60)	3 (20)	—

*Note*: In this table, the embryo yields are summarized for three groups of oocytes: the one fertilized after 1 h from the thawing and the one fertilized after 6 h from the thawing and the control group in which no mechanical measurement was performed.

## 3 DISCUSSION

In this study the variation of the mechanical features during in vitro postovulatory aging of mouse oocytes was investigated by means of AFM‐nanoindentation.

We identified the presence of two, mechanically distinct layers on the mouse ZP characterized by different values of the Young's modulus (*E*1 the outer and *E*2 the inner, respectively), as already observed by our group on human oocytes.^26^ Moreover, also mean values of these two parameters are in the same range even if the distribution is a little bit broader in the case of human oocytes, because of the heterogeneous population of the oocytes retrieved from women and the mechanism of superovulation induction in the mouse that aims to synchronize the oocytes to the same maturation stage. Due to the heterogeneous mesh‐like structure of the ZP surface, the *E*1 values show highly inhomogeneous values from cell to cell, but also within the same cell, just varying by a few micrometers the indentation position. On the contrary, the inner layer is more homogeneous and the results are consistent within the same cell, no matter the indentation position. Hence, we focused on *E*2 to investigate the evolution in time of the oocyte mechanical properties during oocyte in vitro postovulatory aging from the thawing to the occurrence of the visual change due to degradation.

Apoptosis is a well‐known process that begins with oxidative stress and brings to the damage of the mitochondrial DNA, a reduced ATP production and the failure of the mechanisms that regulate the ionic transfer and therefore to the impairment of Ca^2+^ homeostasis.^33^ Ca^2+^ oscillations are different in the aged oocytes both in terms of frequency and amplitude. It was previously suggested that the same process can be read in different ways: in the fertilized oocyte, Ca^2+^ oscillations trigger the meiosis activation and the mechanisms related to the polyspermy block, while in aged oocytes, Ca^2+^ oscillations lead to apoptosis.^3^ The release of Ca^2+^ from the inner cellular compartments is a key process also in the induction of the CG exocytosis, a phenomenon related to the stiffening of the ZP (zona hardening) in the fertilized oocyte. Ovastacin, one of the substances inside the CG, is thought to be responsible for this change in the mechanical properties, as it cleaves ZP2 (one of the three glycoproteins of murine ZP) causing a permanent change in the structure of the fibrils. This is normally a slow process occurring over 2/3 h from fertilization. Evidence of cortical reaction occurrence during oocyte postovulatory aging has already been observed and considered as one of the hallmarks of the aged oocyte.^34^

To prove that indentation measurements do not affect the cell viability inducing a premature aging of the oocytes, the possibility to trigger the CG release with the application of different forces was also investigated. The increase of the fluorescence associated with the CG exocytosis inside the PVS was evaluated during the lifespan of the oocytes, from right after retrieval to the occurrence of visual degradation. The force loading applied up to 20 nN does affect the dynamics of the CG release and we concluded that the mechanical measurement does not interfere with the aging progression.

The evaluation of the variation of *E*2 during the oocyte lifespan highlighted a feature common to all the measurements performed consisting in a statistically significant increase of the ZP stiffness accompanied by a decrease of the viscous dissipation, which is linked to the ZP viscosity, 2 h before any visual change due to degradation. This effect is immediately followed by a ZP softening and recovery of the ZP viscosity as the degradation occurs. In general, when the stiffness of this layer increases, the viscosity decreases, and vice versa.

This suggests the presence of a dynamic cross‐linking of ZP glycoproteins in the aged oocyte. The phenomenon known as “zona hardening” (the increase in the stiffness of the ZP from the mature oocyte to the zygote) has been already reported to occur in hours from a fertilization event,^7^, ^24^ but a reversible and fast reaction occurring after minutes from the sperm entry has only been postulated. Using a very sensitive technique, able to detect small variations related to the mechanical properties of this layered structure, we demonstrated the occurrence and the temporal evolution of an analogous cross‐linking process occurring as a result of the oocyte aging.

To demonstrate that this effect is independent of the adopted measurement strategy, we investigated the stress‐relaxation properties of aging oocytes. To this purpose, we used homemade macroprobes that, when mounted on the AFM head, allow the implementation of microplate rheometric measurements with pN sensitivity. Stress‐relaxation measurements provide one or more relaxation time constants, depending on how many different mechanical contributions are involved. Here, we identified two main players, the ooplasm, and the ZP, whose Young's modulus we measured separately by AFM indentation with and without ZP, finding the former more viscous and softer and the latter less viscous and stiffer.

In this way, we were able to assign the faster time constant to the ZP and the slower one to the ooplasm. The former, which is a measure of the ratio between the ZP viscosity and elasticity, decreases in time during the postovulatory aging, reproducing the behavior previously highlighted with indentation measurements.

The cause of the observed change in the ZP mechanical properties is a spontaneous GC exocytosis as a consequence of oocyte aging, in a mechanism similar to that of the Zona hardening after fertilization. Here, the oocytes were chemically activated with SrCl_2_, a known parthenogenetic activator. It has already been shown that this salt induces a structural change in the oocyte ZP as well as a cortical granule reaction over time.^31^ In the same oocyte, we simultaneously monitored the change in fluorescence intensity related to the CG release and the Young's modulus of the ZP measured by continuous AFM indentation after the addition of SrCl_2_. We observed a sudden increase in the CG‐related fluorescence after the addition of the activator, reaching the maximum intensity over 30/40 min from that point. The dynamics of this induced exocytosis are the same in all the tested cells. Interestingly, also the ZP stiffness increases after the addition of the activator. Nevertheless, the high variability in the *E*2 values does not support a clear triggering effect caused by the cortical reaction on the ZP stiffness increase. One of the reasons for the observed variability could be found in the local nature the GC release induced by SrCl_2_ that is higher in some specific location of the oolemma; as a consequence, also the ZP hardening could significantly vary depending on the position and the measurement; the adopted instrumental approach does not allow to select the measurement point where the exocytosis effect is maximum. A more precise evaluation of the fluorescence limited to the indentation area could be implemented to address this problem.

Other possibilities are related to a dynamic cross‐linking, triggered by some other factors released from the cell, that act in a way similar to ZP1, the ZP glycoprotein that mediates the binding between the fibrils. The detection of Zn^2+^ release within minutes from fertilization together with his role as an important metalloprotein cofactor in the catalytic active site of some enzymes, the variation in ZP fibrils thickness after Zn^2+^ exposure, and the decreasing amount of sperm binding makes this ion one of the possible candidate for this induced variation of the ZP structure and mechanical properties.^31^, ^35^

Finally, we wanted to understand how the observed change in the oocytes mechanical parameters affects the yields of embryo development. For this reason, two groups of oocytes were considered: the “freshly thawed” one was mechanically measured 1 h after the thawing, while the “aged” group was measured after 6 h from the thawing. Immediately after the mechanical measurement oocytes were subjected to an IVF procedure and the embryo yields were evaluated. We show here that the yields of 2‐cell embryos were more than halved in the “aged” group compared to the “freshly thawed” one. And the yields related to the blastocyst development are even lower.

The decreased yields of embryo development in the “aged” sample correlates with the mechanical data as after 6 h from the thawing we can see a statistically significant increase in the oocyte stiffness and a statistically significant decrease in the dissipated viscous energy. This is an important information because we established a direct correlation with the observed change of the mechanical parameters and the yields of in vitro fertilization. Moreover, the yields of embryo development of the “freshly thawed” oocytes previously measured, are very close to the one obtained in the control group. This result further confirm that this method of oocytes analysis does not affect the oocytes quality and probability to be fertilized.

In conclusion, we found out two mechanical parameters (viscosity and elasticity) that can be related to the aging status of the oocyte before any visual feature of degradation can be observed. The measurement of these parameters can be performed with a noninvasive approach which involves the application of forces 6 order of magnitude weaker than those applied in the ICSI procedure.

Moreover, the occurrence of a ZP dynamic cross‐linking in the aged oocyte temporally correlates with cortical granule exocytosis, although we did not address in this paper the molecular mechanism behind this effect, which would require further studies and be the subject of a separate publication.

Other than the investigation of postovulatory aging, the same methodology can be applied to the development of more efficient protocols for oocyte in vitro maturation (IVM), for the screening of drugs able to induce the ovulation. Moreover, ZP mechanical properties might be predictive of oocyte chromosomal abnormalities. Although the clinical use of AFM is limited by the complexity of the experimental setup, its recent evolution is pointing toward automation and easy‐to‐use approaches, so that their implementation in clinics is probably closer than it may appear. Besides, many alternative devices based on force sensing suitable for the use in clinics have been developed in which the experimental setup are much simplified and easy to handle. Moreover, a carefully designed microfluidic system could allow to solve the problem of the oocyte immobilization and sorting, improving the standardization of the clinical procedures. Different devices based on micropipette aspiration and indentation and systems based on hydrodynamic cell stretching have been developed and used for the measurements and sorting of different cellular systems^36^, ^37^ and some of them have also been tested with oocytes.^38^ A possible alternative compatible with the need of preventing oocyte aging and that of the AFM skilled operator in clinics, is a double freeze/thaw approach, in which oocytes are extracted, frozen, transferred to a specialized lab, frozen again and send back in the hospital for a later fertilization and implantation. Although is universally acknowledged that freezing does not affect significantly the fertilization yield, and we have recently demonstrated that freezing do not modify oocytes mechanical properties,^27^ the effect of a double freeze/thaw has not been investigated yet. Further investigations in this field could provide interesting options, that for instance could be used to rationalize the frozen oocyte storage and later use.

Finally, our findings put into evidence that a mechanical essay, based on AFM, represents a very sensitive tool able to detect physiological changes associated with the oocyte status before the occurrence of the visible morphological changes and help to improve the yield of IVF protocols.

## 4 MATERIALS AND METHODS

### 4.1 Oocytes retrieval and sample preparation

MII oocytes were obtained from the oviducts of 7–weeks‐old female mice (C57BL/6) superovulated by intraperitoneal injection of 7.5 IU of PMSG (PG, Italy) followed 48 h later by injection of 7.5 IU of hCG (Merk Life Science, Germany). All experiments were performed under the project #509LAZ19, which was approved by the Administrative Panel of Laboratory Animal Care at the University of Trieste and by the Italian Health Ministry.

Mice were sacrificed by cervical dislocation and oocytes were recovered from ovaries 16 h after the hCG injection into α‐MEM (Sigma) supplemented with bovine serum albumin (BSA) (4%). Cumulus cells were removed by means of a mechanical treatment by pipetting cumulus oocytes complexes with 100 μm inner diameter sized flexipets (Cook medical) in α‐MEM supplemented with 0.03% hyaluronidase (Sigma). Approximately 1 h from the retrieval, oocytes were either immobilized on the surface of petri dishes in α‐MEM supplemented with N‐2‐Hydroxyethylpiperazine‐N′‐2‐ethanesulfonic acid without BSA or frozen according to the protocol described in the supplementary material.

### 4.2 AFM‐indentation measurements

After removing the cumulus cells, oocytes were immobilized on the surface of petri dishes in α‐MEM medium, without BSA, with incubation of 1 h in 5% CO_2_ at 37°C. MII oocytes were selected by visual inspection for the presence of the first PB. Only MII oocytes with a round‐shaped ooplasm, a regular ZP and PVS were considered for the analysis. AFM‐indentation measurements were performed after 1 h from the retrieval with a Nanowizard II AFM (JPK Instruments, Berlin) equipped with an inverted optical microscope. The indenter was obtained by attaching a silica microbead (4.5 μm diameter) to a silicon triangular tipless cantilever of nominal spring constant *k* = 0.32 N/m (Nanoworld with Cr/Au back‐side coating) by using UV sensitive glue (Norland Optical Adhesive 73). Before each measurement, the spring constant of bead‐mounted cantilevers was calibrated by using the thermal noise method, on the surface of glass slides in milli‐Q water. Measurements were performed directly in the petri dishes, on a squared grid of 3 μm × 3 μm at the center of the oocyte. For each point, three force‐distance curves were acquired, for a total of 27 curves for each oocyte. Between one measure and another a recovering time of 5/10 s was used. A maximum force load of 1 nN at a rate of 5 μm/s in z closed‐loop feedback mode was used. The measurement takes no more than 5 min for each cell.

Force distance curves were converted into force‐indentation curves with JPK DP software by subtracting the cantilever bending from the measured height to calculate indentation. The resulting curves were fitted by using a modified Hertz model that considers the contribution of layers with different elasticities. By using this model, two contact points were identified and used to calculate *E*1 and *E*2.^26^ The fitting procedure and the Young's moduli calculations for each oocyte were performed by an Igor procedure. For the viscous dissipation energy calculation, a MATLAB2019 routine was used.

### 4.3 AFM stress‐relaxation measurements

The spring constant of macrocantilevers was calibrated by the spring‐on‐spring method.^30^ The AFM macroprobes used here had a spring constant average value of 1.7 ± 0.4 N/m. Oocytes were immobilized into overhanging support consisting of a plastic coverslip glued on a microscope glass slide, all placed in a plastic petri dish. The sample was located into a drop of medium placed on the border of this stepped coverslip. This setup allowed the side view of the sample through the use of a holder bearing a mirror positioned in front of the cantilever and prevents the crashing of the side‐view mirror on the sample support while performing the measurements. The cantilever was brought into contact with the sample and the petri dish lid containing the support was filled with the medium. At this point, the sample and the cantilever stages were displaced to allow the objective to focus on the mirror and switch, with the help of a side illumination, to obtain a side‐view imaging. The macroprobe was then approached and put in contact with the sample. A CellHesion module that allows to extend the range of the piezo from 15 to 100 μm was used. The sample was compressed of 20 μm from the contact position with a speed of 2 μm/s and a closed‐loop piezoelectric control was used to maintain the deformation constant for 30 s. During this time, the time evolution of force exerted by the cell on the probe (force‐relaxation) was measured. At least five curves per cell were acquired. The force‐relaxation curves can be fitted with a generalized Maxwell model,^30^ to account for the contribution of different layers: the ZP and the ooplasm In agreement with previous studies, two Maxwell elements were sufficient to describe the experimental data.^39^ The equation that describes the model is the following:

$$F\left(t\right)=a0+a1\exp\left(\frac{-\left(t-t0\right)}{\tau 1}\right)+a2\;\exp\left(\frac{-\left(t-t0\right)}{\tau 2}\right)$$

where τ is the relaxation time defines as: 𝜏 = 𝜂 𝐸, where 𝜂 is the viscosity of the sample and *E* the Young's modulus. An Igor procedure was used to fit the data and calculate the relaxation time.

### 4.4 Cortical granule staining

CG exocytosis was evaluated in real‐time after the addition of 25 μg/mL FITC‐LCA (Merck Life Science, Germany) in α‐MEM medium without BSA. The fluorophore was not washed during the experiment, as α‐D mannose is continuously released inside the PVS, and a dynamical staining, proportional to the instantaneous α‐D mannose concentration, is required. Therefore, a high concentration solution of unbound dye should be maintained for the whole duration of the experiment. Images were taken with an inverted microscope (Axiovert 200, Carl Zeiss, Germany), a 40X objective (Zeiss, Switzerland), a DVC camera (Thorlabs) and an X‐Cite fluorescence lamp illuminator (Excelitas Technologies) every hour, with an exposure time of 500 ms. After the image acquisition, the oocyte was put again in dark condition inside the incubator. The overall exposure of the fluorophore to the UV light is less than 10 s for the whole experiment: under this condition, we do not expect a significative fluorophore photobleaching.

Instead, during the analysis of CG‐related fluorescence after the SrCl_2_ addition, images were taken every 5 s for the whole duration of the experiment (90 min) while keeping the UV illumination continuously on. Under this second condition, we evaluated the possible effect of fluorophore photobleaching. Indeed, in a control experiment, shown in Figure 7f, we did not add the parthenogenetic activator and we do not expect a significant variation of the fluorescence. Here, we observed an overall decrease in the fluorescence intensity of about 20%. Several different reasons could account for this decrease, but we can anyway exclude a photobleaching process affecting more than the 20% of the fluorophores over 90 min of exposure, significantly lower than the fluorescence variations observed in Figures 6b and 7a–e.

Analysis of the fluorescence intensity profile associated with the region of the PVS space was performed by a MATLAB routine. The fluorescence intensity profile was drawn and the area underneath the peak corresponding to the PVS was calculated for five different profiles of the same cell for a given time. The background fluorescence was subtracted and the averaged values were plotted.

### 4.5 In vitro fertilization

The 7‐ to 8‐weeks‐old male mice were sacrificed and sperm cells were recovered from the cauda epididymis and transferred in a drop of medium (90 μL/mouse) under paraffin oil. The medium was prepared according to the protocol described by the European Mouse Mutant Archive organization.^40^, ^41^ Briefly, for the sperm cell capacitation, a medium with a high level of CaCl_2_ was used in which 0.1% methyl‐β‐cyclodextrin (Merck Life Science) was added. This molecule, together with Ca^2+^ are needed for the sperm hyperactivation that consists in the change in sperm motility from straight trajectories to circular ones.^42^ Sperm cells are incubated for 1 h in this medium. In the meanwhile, the drop with the cumulus cells in culture was took and the medium was substituted with 100 μL of the fertilization medium (CARD medium, Cosmo Bio).^43^ Oocytes were thawed and measured by AFM‐indentation about 1 and 6 h (in the case of the aged sample) from the thawing, except from the control sample in which we performed exactly the same procedure with the exception of the AFM indentation. After, oocytes were put in the fertilization drop and 5 μL of sperm suspension was added. The results coming from two different experiments (6–8 oocytes for each) were then merged as the embryo yields were comparable. In this way a total of 14–15 oocytes were measured for each condition.

After the addition of the gametes, the fertilization drop is stored in the incubator at 37°C, 5% CO_2_.

After 3 h oocytes are removed from the droplet and moved to a 100‐μL drop of HTF medium (Cosmo Bio) without reduced glutathione in order to remove the excess of sperm cells and the cell debris. The development of 2‐cell embryos was evaluated 24 h later by visual inspection and the embryos were moved to a 100‐μL drop of KSOM Embryo Medium (Merck Life Science). Forty‐eight hours later the development of the 8‐cell stage embryo was evaluated by visual inspection, and 8 h later, the early blastocyst was observed and, about 15 h later, few of the embryos reached the late blastocyst stage.

### 4.6 Statistical analysis

The statistical difference between the samples at different times was evaluated by using the one‐way Anova parametric statistical test with GraphPad Prism 5.0. A *p* value <0.05 was considered to be statistically significant. For the mechanical data related to postovulatory aging of fresh and thawed oocytes (Supporting Information S4), the nonparametric Kruskall–Wallis test was performed considering a *p* value <0.05 statistically significant, while for the data of the oocytes subjected to in vitro fertilization procedure, the nonparametric Mann–Whitney test was used considering a *p* value <0.05 statistically significant.

## AUTHOR CONTRIBUTIONS

**Alice Battistella:** Formal analysis (lead); investigation (lead); methodology (lead); validation (equal); visualization (equal); writing – original draft (lead); writing – review and editing (equal). **Laura Andolfi:** Data curation (supporting); methodology (supporting); project administration (equal); supervision (supporting); validation (equal); visualization (equal); writing – review and editing (equal). **Michele Zanetti:** Investigation (equal); methodology (equal); visualization (equal); writing – review and editing (equal). **Simone Dal Zilio:** Data curation (equal); methodology (equal); validation (equal); visualization (equal); writing – review and editing (equal). **Marco Stebel:** Methodology (supporting); writing – review and editing (equal). **Giuseppe Ricci:** Funding acquisition (supporting); resources (supporting); validation (equal); visualization (equal); writing – review and editing (equal). **Marco Lazzarino:** Conceptualization (equal); funding acquisition (lead); methodology (supporting); project administration (supporting); supervision (lead); validation (equal); visualization (equal); writing – review and editing (lead).

## CONFLICT OF INTERESTS

The authors declare no competing financial interests.

5

### PEER REVIEW

The peer review history for this article is available at https://publons.com/publon/10.1002/btm2.10294.

## Supporting information


**Appendix**S1: Supporting Information
