# Supplementary material for: Atomic force spectroscopy‐based essay to evaluate oocyte postovulatory aging
Source: Bioeng Transl Med. 2022 Jun 9;7(3):e10294. doi: 10.1002/btm2.10294 (PMC9472013; doi:10.1002/btm2.10294)
Supplement: Supplementary file 1 — AppendixS1: Supporting Information [file BTM2-7-e10294-s001.pdf]

SUPPLEMENTAL MATERIAL

Atomic force spectroscopy-based essay to evaluate oocyte post-ovulatory ageing

Alice Battistella [1,2], Laura Andolfi [1], Michele Zanetti [1,2], Simone dal Zilio[1], Marco Stebel [2], Giuseppe Ricci[3], Marco Lazzarino [1]

[1] CNR-IOM SS 14 km 163.5 Area Science Park Basovizza 34149 Trieste Italy

[2] University of Trieste, P. le Europa 1 34100 Trieste Italy

[3] IRCSS Burlo Garofalo, strada dell'Istria 34100 Trieste Italy

Materials and methods

SEM preparation

Oocytes, attached to plastic coverslips were fixed in 4% paraformaldehyde in PBS, postfixed with 1% osmium tetroxide and dehydrated with an increasing concentration of ethanol prior to critical-point drying with carbon dioxide.

Oocyte freezing and thawing procedure

After the removal of the cumulus cells, oocytes were transferred, by the help of micropipettes for the manipulation, into a drop of α-MEM supplemented with 20% FBS and incubated at 37°C 5% CO_2 for 30min. The incubation with the serum allows to avoid damages to the ZP during the freezing procedure.
In the meanwhile, cryogenic tubes were put inside a box with ice and for each one 5 µl of PB1 supplemented with 1M DMSO were added (1). There, oocytes are transferred to the same 1M DMSO solution. It is important to stress here that this is a very delicate step and everything has to be done very fast to avoid the oocytes degradation. Oocytes are transferred to the cryovials (5 oocytes/vial) with the micropipettes and left in ice for 5/10 minutes. After that, 45 µl of cryoprotective agent (DAP213) were added for each vial and left equilibrate again for 5/10 minutes before plunging in liquid nitrogen (LN). The procedure is shown in Figure 61 (2).
For oocytes thawing, 0.25M sucrose solution in PB1 was prepared and warmed before use. At the same time 2 ml of fresh medium (α-MEM with 4% BSA) were warmed in a 35 mm petri dish. A vial is removed from

liquid nitrogen and, after LN discarding, 1.5 ml of thawing solution was put inside, by gently pipetting the sample was dissolved and the drop transferred to a petri dish. Also, this step is very delicate and has to be performed very quickly in order to avoid oocyte degradation. Here, with the help of manipulation pipette, oocytes are transferred to the petri dish with the α-MEM medium inside and left to recover in the incubator for at least 30-40 minutes.

ZP removal

Oocytes were transferred to a drop of acid Tyrode's solution (3) and mechanically pipetted with micropipettes having a capillary diameter of 100-150μm. This step has to be performed quickly (less than 10 s) in order to avoid the collapse of the ooplasm. Oocytes were then transferred to a second drop of medium with BSA and left equilibrate for at least 15 min before performing the measurement. This procedure does not affect the oocyte quality as it has been optimized to implement the rate of the implantation after the fertilization of zona-free oocytes (4).

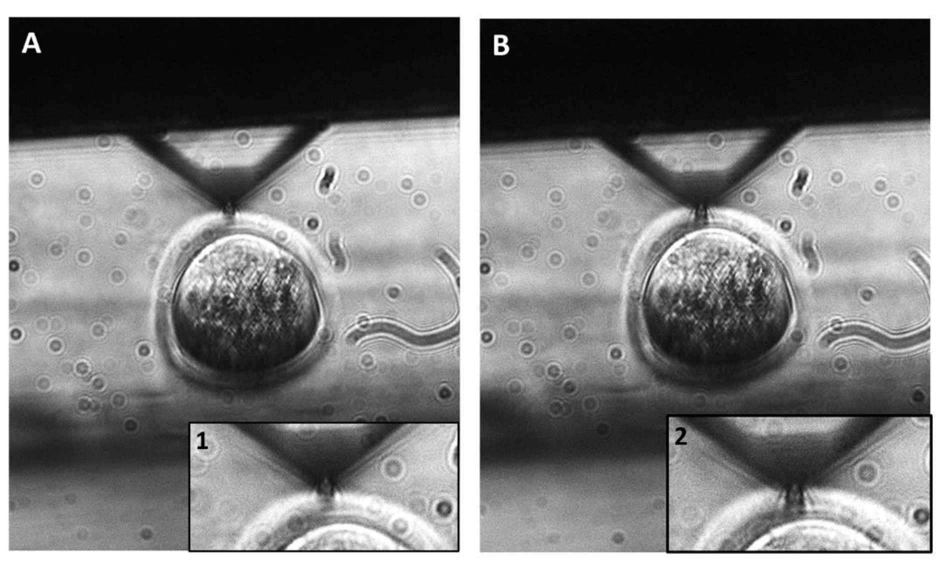

Fig. S1: Side view images of an oocyte indented by a bead-mounted triangular cantilever. The position at static contact (A, 1 zoomed) and the maximum indentation depth (B, 2 zoomed) is shown: no deformation of the PVS and ooplasm is observed.

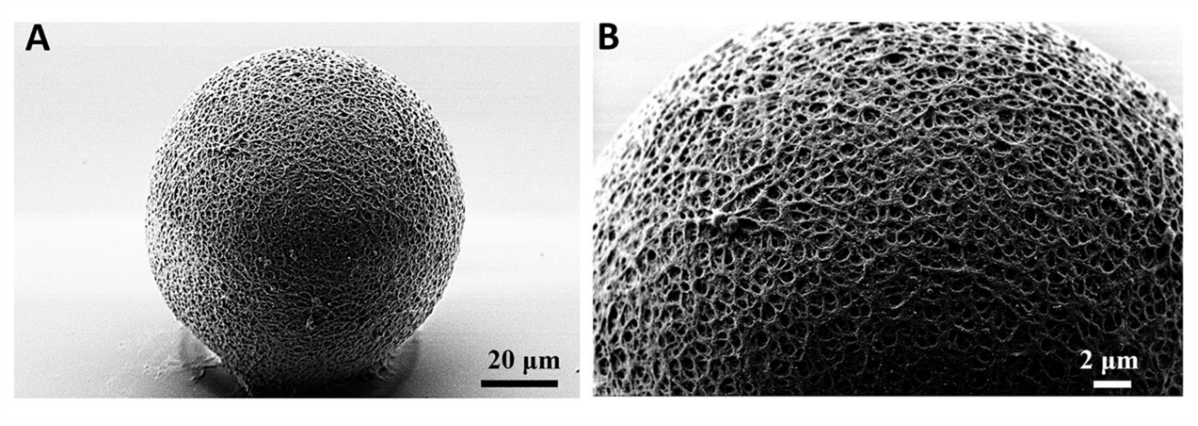

Fig. S2: **SEM micrographs of a murine oocyte.** (A,B) different magnification of the zona pellucida showing the spongy, mesh-like structure of this layer

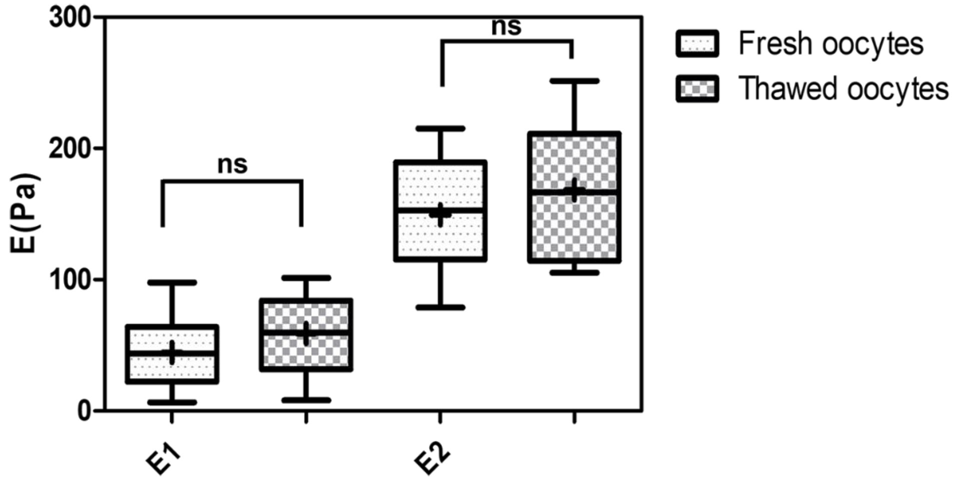

Fig. S3: Distribution of E1 and E2 values for fresh and thawed oocytes. The values inside the box-whisker plots represent the first (25%) and third quartile (75%), the (-) indicate the maximum and minimum observations; the line within the box represents the median value (50%); while the mean value is indicated in the plot as (+). One way anova parametric statistical test was performed, significance level was set at p values <0.05. No statistically significative difference was detected between the mean values of the groups analyzed (12 mice, N=20 for each condition).

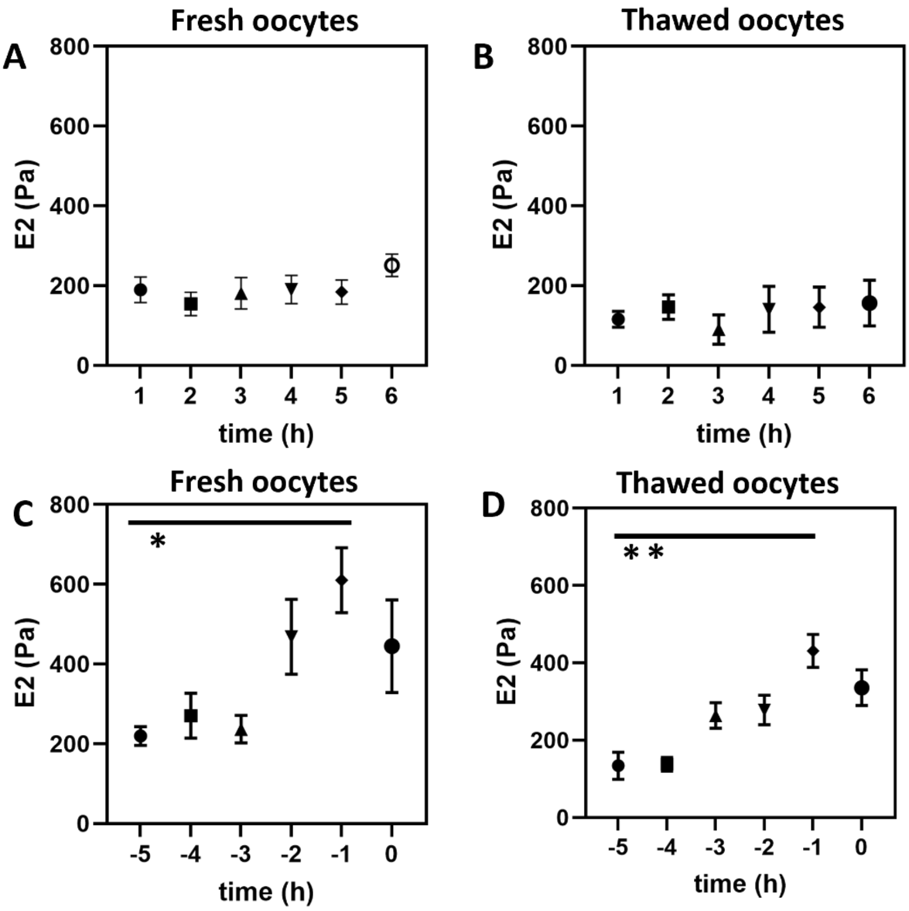

Fig. S4: E2 change during post-ovulatory ageing for fresh and thawed oocytes. E2 change during in vitro post-ovulatory ageing of fresh oocytes (**N=6**) (A) and thawed oocytes (**N=9**) (B) that did not reach degradation during the observation period measured up to six hours from the retrieval/thawing.
E2 change is showed during in vitro ageing for fresh oocytes (**N=6**) (C) and thawed oocytes(**N=14**) (D)that reached degradation during the observation period measured up to 6 hours from the retrieval/thawing. A significative increase in ZP stiffness is observed one hour before the visual degradation in both fresh and thawed oocytes. Data of each cell are aligned on the time of degradation and plotted against the time scale of the time before degradation. The significance level was set at *$p < 0.05$, **$p < 0.005$

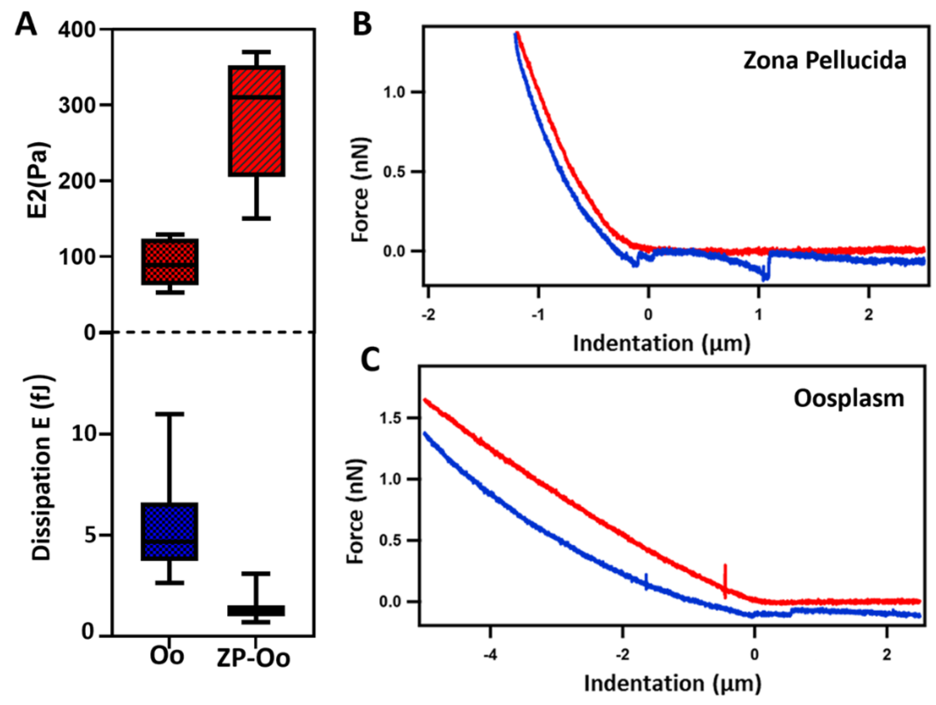

Fig. S5: **Different mechanical properties of ooplasm and zona pellucida.** (A) Mechanical characterization of Ooplasm (Oo) and oocytes surrounded by ZP (ZP-Oo), oosplasm is characterized by an higher dissipation energy (in blue) and a significantly lower stiffness (in red) compared to ZP, (B,C) this is highlighted by the force-distance curves shown on the right.

References

1. Nakao, K., Nakagata, N., and Katsuki, M. Simple and efficient vitrification procedure for cryopreservation of mouse embryos. *Experimental animals* 1997; 46: 231-234.

2. Nakagata, N., Takeo, T., Fukumoto, K., Kondo, T., Haruguchi, Y., Takeshita, Y., Nakamuta, Y., Matsunaga, H., Tsuchiyama, S., Ishizuka Y., Araki, K. Applications of cryopreserved unfertilized mouse oocytes for in vitro fertilization. *Cryobiology* 2013; 67: 188-192.

3. Nagy, A., Gertsenstein, M., Vintersten, K., Behringer, R., Removal of Zona Pellucida. *CSH Protoc.* 2006*;* 3

4. Jelinkova, L., Pavelkova, J., Strehler, E., Paulus, W., Zivny, J., Sterzik, K. Improved implantation rate after chemical removal of the zona pellucida. *Fertility and sterility* 2003; 79: 1299-1303.
